# ‘I am a father but not pregnant’: a qualitative analysis of the perspectives of pregnant couples on male partner role during pregnancy care in Bamenda, Cameroon

**DOI:** 10.1186/s12978-024-01928-5

**Published:** 2024-12-23

**Authors:** Lily Haritu Foglabenchi, Heidi Stöckl, Tanya Marchant

**Affiliations:** 1 Department of Disease Control, London School of Hygiene and Tropical Medicine, Keppel St, London, WC1E 7HT UK; 2 Maternal and Child Health Unit, Cameroon Baptist Convention Health Services, Finance Junction, P.O Box 1, Bamenda, North West Region Cameroon; 3 Institute for Medical Information Processing, Biometry and Epidemiology, Faculty of Medicine, Ludwig-Maximilians-Universität München, Munich, Germany

## Abstract

**Background:**

The reduction of maternal mortality has stagnated globally. Estimates project a rise to 140.9 deaths per 100,000 live births by 2030, which is double the Sustainable Development Goal target. Male involvement in pregnancy care has been proposed as an intervention to improve maternal and child health outcomes. However, there is limited understanding of how communities view the role of men beyond the instrumentalist approach that only targets men as accompanying partners without altering the underlying gender and socio-cultural determinants that shape their involvement in pregnancy care. This study broadens existing research by exploring and and contextualising the role of male partners during pregnancy in Bamenda, Cameroon.

**Methods:**

This study employed a qualitative design underpinned by symbolic interactionism. We conducted 68 semi-structured interviews (SSIs) and three focus group discussions (FGDs) with purposively selected pregnant women (n = 38 SSIs; n = 2, FGD) and male partners (n = 30 SSIs; n = 1, FGD) in an urban hospital in the North West Regional capital—Bamenda. Nvivo was used for data management and subsequently, we performed thematic analysis using a critical discourse lens to generate manifest and latent interpretations of study findings.

**Results:**

The role of male partners reflected hegemonic masculinity and was broadly conceptualised in three categories: breadwinner, protector/comforter, and ‘sender’ for antenatal care. Perceptions of men’s role differed between male and female participants. While women sought male involvement for pragmatic reasons like joint attendance of antenatal care, psychosocial support (affirmation) and assistance with domestic chores, men limited their involvement to roles that matched gendered preconceptions of masculinity like financial support for antenatal fees, maternal nutrition and birth supplies. Nonetheless, the perceived benefits for antenatal attendance was expressed by some men in terms of the direct access it gives them to pregnancy-related education from experts, paternal bonding and the appeal of fast-track services for couples.

**Conclusion:**

Male involvement in maternal and child health in Bamenda Health District is an extension and reflection of how patriarchal norms on masculinity are constructed and adapted in this setting. To address gaps in male involvement, intervention designers and implementers will need to take into account prevailing culture-specific norms while deconstructing and leveraging masculine ideals to situate male involvement in the prenatal context.

## Background

Pregnancy and childbirth in African settings has traditionally been treated as a domain that only pertains to women, although human reproduction involves men and women. During the 1994 International Conference on Population and Development (ICPD), the need for male involvement in reproductive, maternal and child health was emphasised [1]. The ICPD agenda specifically called for men’s shared responsibility in parenthood, sexual and reproductive health, HIV prevention and support for female autonomy in matters that pertain to their health and wellbeing [1]. This call to action was underpinned by the recognition that men could act as allies—not only as equal partners and responsible parents but co-beneficiaries with significant influence on maternal and child health outcomes [2–5]. In response to the ICPD agenda, the last three decades has witnessed an interest in the role of men in health programs that have traditionally been women-centred.

Several studies and systematic reviews have documented the benefits of male involvement in reproductive, maternal, newborn and child health outcomes. As breadwinners and traditional heads of households, men have the opportunity to provide monetary, psychosocial and practical support that can overcome demand-side barriers to maternal and child health services. Evidence suggests that male involvement is associated with improved skilled-birth and post-natal care attendance [6–8]; contraceptive uptake and birth spacing initiatives [9–11]; reduction in maternal burden associated with pregnancy [12]; facilitates spousal communication during pregnancy [6] and improves utilisation of antenatal and HIV prevention services [13–15]. Additionally, male partner participation in maternal and child health is associated with improved maternal nutrition, uptake of childhood immunization and promotes infant-feeding practices [6, 15]. Despite these benefits and the emerging evidence that men are ‘gate-keepers’ who determine if and when women access health services, the conceptualization of their role during pregnancy is often grounded in westernized and biomedical approaches that fail to account for contextual variations in the nature and extent of their involvment in pregnancy care [17]. This Euro-American conceptualization of male involvment that antagonize expectant fathers and view their absence in globally assigned indices as nonchalance could could likely be a crucial factor accounting for sub-optimal or what Powis calls context-independent interventions that reinvent rather than move the goal post [18] on Sustainable Development Goals 3.1 and 5.6: to reduce the global maternal mortality ratio to less than 70 per 100,000 live births by 2030; and ensure universal access to sexual and reproductive health as agreed in the ICPD agenda [16].

Understanding how communities frame and conceptualise expectant father's role and behaviours during pregnancy is imperative in developing interventions to address gaps in their involvement in maternal and child health. Historically, most studies on male involvement have focused on documenting the epidemiological prevalence and correlates of male involvement mainly from the perspectives of women [13, 19, 20]. This has reinforced the peripheral position of men while failing to adequately represent their gendered perspectives during pregnancy, and broadly, the need for and value to be derived from their involvement in programs that have traditionally targeted women [21, 22]. It also overlooks the fact that fathers are men first and exist within contextual constructs of masculinity that need to be examined and leveraged in the development of interventions that seek to improve their health and that of their families. To this aim, we conceptualised our study with the assumption that irrespective of the line of enquiry on male involvement, the question of how men define and value their role in maternal health is fundamentally underpinned by gender—particularly cultural identity and ideology on masculinity.

According to Connell, masculinity is a socially constructed role or gendered identity that men uphold to distinguish themselves from other men while maintaining dominance over women [23]. It is not fixed but operates within a spectrum of interrelated forms—with the dominant form called hegemonic masculinity [23, 24]. Hegemonic masculinity is the prevailing form of masculinity across Cameroon and more so in the North West Region where this study was conducted. In this context like in most traditional African societies, men maintain their dominant status over women by pursuing breadwinning and social protection roles, ultimate decision-making, reproduction to secure their lineage and hesitancy to venture into maternal spaces [25–28]. This study therefore draws on Connell’s conceptualisation of masculinity to contextualise the perceived role of men during pregnancy and assesses their perceived need for involvement in maternal and child health in the North West Region of Cameroon.

## Methodology

Our study was part of a formative project [29] involving qualitative methods, semi-structured interviews (SSIs) and focus group discussions (FGDs) that took place between July and December 2021 in Bamenda Health District, Cameroon. Our methods and study findings are reported in line with the Consolidated Criteria for Reporting Qualitative Research (COREQ) [30].

### Study design

We employed a qualitative research design informed by the social constructivist paradigm [31] and underpinned by symbolic interactionism—an approach that focuses on the construction of meaning within wider social and cultural settings [32]. This approach enabled us to move beyond mere descriptions and conceptually understand how pregnant couples construct meaning and attribute value to the role and involvement of men in antenatal care in Bamenda, Cameroon.

### Study setting

Bamenda Health District is situated in an urban and semi-urban area that hosts the main referral hospital in the North West Region (Bamenda Regional Hospital), 17 health areas and 35 health facilities—with an estimated population of 800,000 inhabitants [33]. Our study participants were drawn from Nkwen Baptist Hospital—a semi-urban, faith-based hospital run by the Cameroon Baptist Convention Health Services. The hospital is the largest provider of health care in the city on a par to the level 2 Bamenda Regional Hospital with a 114-bed capacity and high-volume antenatal clinic that attends to over 400 pregnant women per month [34]. The antenatal clinic provides routine services for couple HIV testing and has made attempts to fast-track services for men who accompany their partners for antenatal care.

The majority of residents in Bamenda Health District are ethnically ‘Graffi’ (a contracted name for Grassfields) with internal migration presenting an increasing mix of cultural representation from across Cameroon and neighbouring Nigeria [35]. Grassfield communities who trace their ethnic identity to the Ngambe-Tikar people of the northeastern part of the North West Region have historically been patrilineal [35, 36]. While the traditional hierarchy in the Grassfield affords women certain autonomies and disproportionately regards pregnancy and childbirth as a female domain, it is often influenced by men’s performance of masculinities [28, 36, 37].

### Participants

This study comprised of 88 participants of whom 36 were men (n = 30 SSIs; n = 6, 1 FGD) and 52 were women (n = 38 SSIs; n = 14, 2 FGD) who were either pregnant or early postpartum at the time of the study. Any man with a pregnant or recently post-partum partner (within two years) over the age of 18 was included in the study.

### Sampling and recruitment

The approach for participant selection was purposive maximum variation sampling as we sought to capture a broad range of perspectives on the phenomenon of inquiry [38]. Sampling was assumed to be complete once no new data or theme emerged [39].

Our recruitment strategy was informed by the literature on male involvement in maternal and child health and our observation of women/men who presented for ANC and infant welfare clinics [19]. We began with an announcement about the study during group antenatal or postnatal care at the hospital. With the assistance of the lead nurse, we observed antenatal/postnatal care and approached potentially suitable participants for assessment and consent. All men in attendance were approached given their limited numbers while men who were not in attendance were recruited through pregnant women who shared the phone numbers of their partners for a direct call. Of the 93 eligible study participants we approached, 88 consented.

### Data collection

Focus group discussions and semi-structured interviews were used in parallel to collect data. This resulted in an iterative process where concepts noted in one method were further explored or confirmed in its counterpart method [40, 41]. Initially, we conducted 21 SSIs and used these to refine topical themes, examine individual perspectives and explore sensitive topics that would otherwise not have been possible during group discussions [41]. This was followed by the two female FGD and one male FGD each comprising of 6-8 members where we broadly explored community perspectives on the socio-cultural constructs that underpin male partners’ roles during pregnancy. This approach was based on the evidence that group settings are well suited for generating shared meanings and representation on gender and health [40, 42]. As both methods were used in tandem, FGDs also afforded us the opportunity to identify pertinent themes for in-depth exploration during subsequent (n = 47) individual interviews.

Data collection was led by the lead author who is a native of Bamenda and was assisted by two research participants—a Nurse Midwife (NN) and Sociologist (MT)who were trained in qualitative methodologies. Open-ended thematic guides were used for SSIs and FGDs. These data collection instruments were pretested with one pregnant and two postpartum women. These featured flexible, broad and free-flowing questions that explored perspectives and normative behaviours around male role during pregnancy; and perspectives on the need/ motivations for male ANC attendance.

Data collection was conducted in both English and Pidgin language within the clinic premises and occasionally in the community with men who could not travel to the clinic. SSIs sessions lasted between 22 and 56 min while and FGDs sessions lasted between 1hr14mins and 2 h. Both sessions were audio recorded with permission from participants. The lead investigator held debriefing meetings after sessions with research assistants and her supervisory team to explore preliminary insights and develop analytic memos.

### Data analysis

Interview and group discussion data were translated (where applicable) and transcribed verbatim. Following transcript and quality checks, our data were organised, uploaded and coded using NVIVO software [43]. In line with the constructivist design of this study, data analysis was iterative and we employed the 6-step thematic framework analysis approach by Braun and Clarke to explore perceptions on male roles and need for involvement during pregnancy [44]. This involved: data familiarisation through reading and re-reading of transcripts; collaborative coding of initial codes both deductively and inductively by LHF and NN; theme identification, review, integration by LHF, TM and HS and interpretation. Once identified, themes were further analysed using a hegemonic lens through a Critical Discourse Analysis (CDA) [45, 46] that characterised male roles and motivations for involvement in pregnancy care. We specifically analysed emerging themes to uncover latent and manifest narratives that sustain gender power relations and reflect hegemonic ideals on male role and involvement in the prenatal context [45]. In addition, we explored how participants navigate and reconcile discordant views on the perceived role of men and their involvement in a setting that has traditionally been viewed as the preserve of women.

### Ethical consideration

The London School of Hygiene and Tropical Medicine in the United Kingdom reviewed and provided ethical clearance for this study (Ref: 18003). Locally, the Cameroon Baptist Convention Health Services Institutional Review Board also provided clearance (IRB2019-33). Prior to participation, written informed consent was obtained from all participants.

## Results

Table 1 outlines the demographic characteristics of our sample. Of the 52 pregnant and postpartum women we interviewed, 37 (71%) were married, 13 (25%) were cohabiting while 2 (4%) were single. Majority (84%) reported being in professional or self-employment and all had formal education with one third having college or university level education. Our male participants had broadly the same characteristics as female participants with the exception of employment type—the majority of men were self-employed as bike or taxi drivers, farmers, traders and vocational workers. Very few of the men we interviewed (19%) reported that they had attended antenatal care with their partners.

**Table 1:** Participant demographics

Participant demographics	SSIs (n = 68)		FGDs (n = 20)	
	# Women	# Men	# Women	# Men
# of participants	38	30	14	6
Mean age	30		31	
Marital status				
Married	29	20	8	5
Cohabiting	7	10	6	1
Single	2	0	0	0
Level of education				
Primary	10	7	6	2
Secondary-high school	13	11	7	3
University or polytechnic	15	12	3	1
Employment status				
Employed professional	15	7	6	4
Self-employed	16	23	7	2
Not employed	8	0	0	0
ANC attendance with partner	4	7	6	2

Two overarching domains emerged from the SSIs and FGDs: (A) perspectives on male roles during pregnancy and (B) perceived need for male ANC attendance. We identified three themes under male role during pregnancy: (A1) The provider, (A2) The protector and comforter (physical & psychosocial support) and (A3) The ‘sender’ for ANC attendance. Perceived need for male involvement in ANC attendance included four themes: (B1) direct access to pregnancy education and accountability for medical compliance, (B2) Paternal bonding and shared responsibility (B3) reputational and social protection, and (B4) fast-track services for couples.

**Fig. 1:**
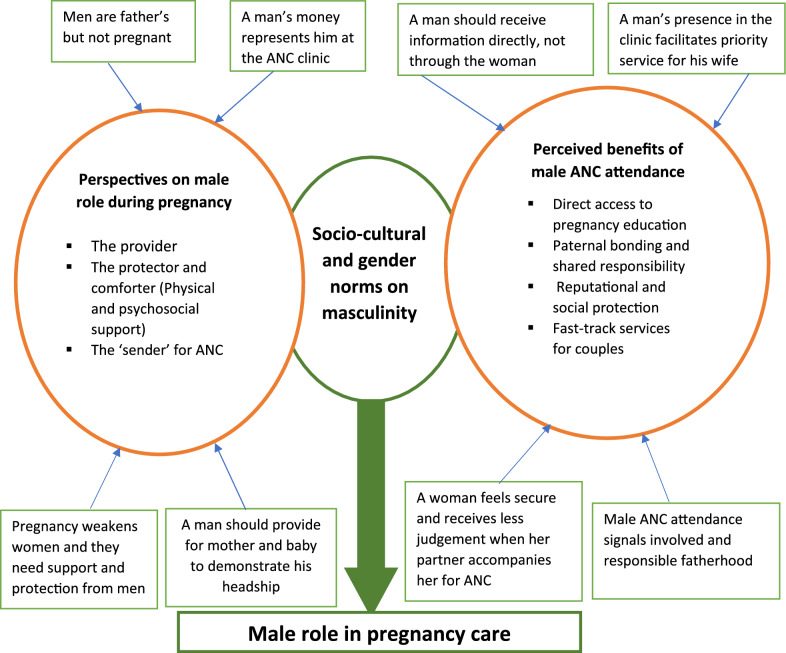
Conceptualisation of pregnant couple perspectives on male role and need for involvement during pregnancy care in Bamenda, Cameroon

Figure 1 provides a conceptual overview of the themes and factors that underpin perceptions on male role during pregnancy and need for ANC attendance.

## Perspectives on male role during pregnancy

This theme unearths the relationship between masculinity, fatherhood and male role in maternal and child health in Bamenda. Our analysis revealed that the construction of masculinity reflects hegemonic ideals that directly influence the perceived role of men during pregnancy in the Grassfield. This was evidenced by three key sub-themes: the breadwinner (for maternal nutrition, ANC fees and birth preparedness), the protector and comforter—physical support (domestic chores), psychosocial support (comfort & patience), and the ‘sender’ for ANC. These are explained below with illustrative quotes.

### The provider

Nearly all participants reported that men were expected to assume financial responsibility for their partner’s pregnancy by providing food, paying for antenatal care and overseeing the purchase of clothing and birth preparedness supplies for mothers and babies. Providing for maternal nutrition was the most cited item under the breadwinner role, followed by fees for antenatal care and birth supplies for mothers and babies.

*“Women need special food during pregnancy. A man’s responsibility is provision for good nutritional intake for the sake of her and that of the baby”.* (Male FGD participant, early 30s)

Despite the majority of participants mentioning that breadwinning was the expected role of men, this was not strongly emphasided by female participants. Particularly, male participants emphatically reported that the breadwinning role was obligated and supersedes all roles because fathering a child affords men cultural respect and mandates them to uphold their headship through financial provision.

*“The financial role to me does not need to be stressed because it is the man’s responsibility. As we Bamenda (Grassfield) believe, once you get into the business of getting a wife or making her pregnant, you are considered a man. That perception comes with a lot of respect and responsibilities. To maintain your respect as a man and leader, you should engage in the activities of men and be financially stable”*. (Male FDG participant, late 20s)

Participants equally reiterated that the provider role is so ingrained within the Cameroonian context to the extent that a man who makes a woman pregnant out of wedlock, could attract legal action for him to assume financial responsibility for the pregnancy.

*“Contextually we live in a society that is highly masculine and already places the demand on the man to take responsibility for pregnancy. And I think the emphasis around this is so strong to the extent that when a man is in an illicit or premarital union with a lady and gets her pregnant, her parents go to the extent of summoning him to the police or tracking him down to undertake his responsibilities”.* (Male FDG participant, mid 30s)

While some participants acknowledged that pregnant women could equally support themselves, it was saliently stated that the provider role was the culturally sanctioned role for men to lead.

*“Here is the truth: if a man wants a happy home, he is supposed to afford the bills. It does not matter how we toast it. Even if a woman works or not, a man is expected to shoulder all the responsibility for providing. We are Africans”* (Male SSI participant, mid 30s)

### The protector and comforter (physical & psychosocial support)

In addition to the breadwinning role, participants frequently emphasised that pregnancy could be distressing for women. As such, being a man mandated the assumption of supportive roles to protect the physical and psychosocial wellbeing of the pregnant partner. Physical support highlighted by participants included domestic chores and errands while psychosocial support included positive affirmations, patience (with mood swings) and comfort.

*The man needs to support with house chores like cooking, washing dishes and dresses etc. You need to assist them because we believe at that during these periods, they are not strong enough and should not be stressed (*Male FGD participant, late 20s)

In light of psychosocial support, a female respondent noted:

*“When I am pregnant, my system changes. I become weak and need support from him till the day I put to birth*.” (Female SSI participant, mid 30s)

We noted that male participants did not echo psychosocial support as strongly as female participants who reported that some men limit their responsibilities only to the provision of finances at the expense of psychosocial support.

*“Some men feel like their responsibility is only providing for the baby’s needs and the mother’s. They don’t care about the moral, or emotional part of it”*. (Female SSI participant, late 20s)

During the female FGD in particular, participants opined that men don’t often support with domestic chores and the lack of psychosocial support from men could likely be the cause of health complications women experience during pregnancy.

*“…When you ask for help, he will think that you are just looking for an excuse to relax and some women are being cautioned in the hospital about overworking during pregnancy” (*Female FGD participant, late 30s)

*“A man needs to give her all the support she needs because there are times the woman develops complications from pregnancy. It comes as a result of stress. Normally if a husband fails to support the wife, she might end up with preterm labour”.* (Female FGD participant, early 20s)

### The ‘sender’ for ANC attendance

Across SSIs and FGDs, male antenatal attendance did not feature as a key role until the moderator enquired. While the majority of female participants unanimously affirmed male ANC attendance upon probing as an important role, most male participants intimated that their role as co-authors of pregnancy did not fully extend to ANC attendance. They argued that they are not pregnant albeit fathers. They conceptualised their involvement in antenatal care as act of ‘sending’—a representation of their authority and influence over the bearers of pregnancy. Some participants echoed this as a form of delineating gender roles between men and women.

*“I am a father, but I am not pregnant. Attending ANC is not my duty. We have defined duties for everyone in this culture. A woman goes for antenatal, I afford the bills and send her for as many ANC classes as possible”*. Male SSI participant, early 40s)

Female participants noted that men limit their role to financial provision and assume the finances they give women for antenatal care represents them at antenatal clinics.

*Once a woman is given money and sent for antenatal, men assume their money represents them and they have done their job*. (Female SSI participant, early 30s)

They however argued that male antenatal attendance was a key role in pregnancy because marriage and parenthood call for shared responsibility and oneness without the limitation of ANC attendance to women alone.

*Of course! Antenatal attendance is a man’s duty because the child is not just the woman’s own. You both are into it and so, if we can do it together, the better* (Female SSI participant, mid 30s)

Some added that, since men generally receive recognition once a baby is born, attending ANC for the pregnancy was imperative.

*ANC attendance is part of men’s roles although women keep coming and at the end, it is usually the men who are recognized for babies. Not even the women who carried the babies in their womb* (Female SSI participant, late 20s)

## Perceived benefits of male ANC attendance

Despite the aforementioned narrative that limits male involvement in ANC to the ‘sender’ role, some respondents challenged this and reported that there are shifting mindsets on the role of men in antenatal care. As outlined below, this was reflected in four sub-themes on the perceived benefits participants advanced for male ANC attendance:

### Direct access to pregnancy education

Across all categories of respondents, accountability and direct access to expert MCH information were the most cited motivations for male ANC attendance. Male participants in particular intimated that they were uncomfortable with female-led post ANC discussions and preferred accessing information directly from health providers.

*Most of the time when she goes for ANC and comes back talking about what happened during her ANC visit, I don’t pay attention. I don’t know why but I am not comfortable with that discussion*. (Male SSIs participant, late 30s)

*Attending ANC feels like a class of its own. In my case, I was listening and she was listening. With that, I was educated and when she says something, it is no longer ‘strange’ to me*. (Male SSIs participant, mid 30s)

A similar view was confirmed by female respondents who noted that male discomfort with female-led ANC information stemmed from gender stereotypes that classify women as voiceless with no authority to instruct men. They also noted that men abide to or respect health education and recommendations when it comes directly from the health facility than when it is relayed by female partners.

*Most men believe a woman has no voice—her place is in the kitchen and should not instruct men on their responsibilities. Attending ANC gives them access to the Doctor/Nurse who tells them what they need to do directly—they take it seriously than when we take this information home. It might lead to an argument and endanger our lives* (Female SSIs participant, mid 20s)

Another participant noted that post ANC discussions tend to be a source of conflict between couples.

*Most of the time when she is talking about what happened during her ANC visit, I’m not paying attention. I don’t concentrate on what she is saying and will usually tell her that “I am tired, I just returned from work. We will talk later”…and this usually brings problems* (Male SSIs participant, late 30s)

In this same vein, male respondents reported in several instances that they were accountable for the health of their pregnant partners and attempted to demonstrate their responsibility/leadership by attending ANC attendance to ensure women adhere to medical recommendations. This was echoed by female participants who reported that they rely on the support of their male partners to reinforce and hold them accountable on pregnancy related education.

*There are times when we come here and feel dizzy or even doze off. So when a man accompanies the wife, he can listen to lectures on our behalf and when we get home, they can help us with the information*. (Female FGD participant, late 20s)

### Paternal bonding and shared responsibility

Among male participants, ANC attendance was perceived as a form of male involvement in father-baby bonding in view of their prospective roles as fathers. Female respondents sought the company of their partners at ANC for spousal bonding and shared responsibility in the burden of pregnancy. As noted by this male respondent, male ANC attendance demonstrates responsible and involved fatherhood from the early stages of life.

*As a potential father who takes his pregnant wife for ANC, it creates a child-father connection because even a child in the womb is already informed that you are a present father* (Male SSIs participant, mid 30s)

Similarly, another participant added that the prospect of seeing one’s baby or listening to their heartbeat during echography was a special moment for bonding.

*When a man attend’s ANC, he get to share in his baby’s heartbeat. During echography, you see your child. These are all great moments for a father* (Male SSIs participant, early 30s)

Among female respondents, the value for male ANC attendance was perceived as a form of companionship that lifts burdens off women and strengthens spousal relationships.

*When a man accompanies his wife for ANC, it gives her a sense of companionship that she is not in it alone. He shares the burden and makes her feel secure that she has someone she can turn to* (Female SSIs Participant, late 20s)

### Social and reputational protection

In a context where the social and reputational status of women is derived from men, most female participants reported that male ANC attendance comes across as a form of reputational protection, a special treat and show of love.

As suggested below, a woman feels safe and protected when a man accompanies her for ANC.

*It just feels good to come for ANC with a husband because I am young and when people see me with a pregnancy in the clinic, they might wonder what a child is doing here. When he comes with me, I feel confident*. (Female SSIs participant, early 20s)

While female respondents associated male ANC attendance with a special status and an expression of love, male participants did not perceive it as such.

*From my point of view, a woman might feel special and see a man accompanying his wife for ANC as proof to the society that he loves her but for the man, he will not see it that way* (Male SSIs participant, late 30s)

*I know that it would be very exciting for a woman to have her husband come with her. This will make her feel that she has a husband that is really taking care of her. If he comes at least once it will be a very good thing and the wife will be very happy*. (Female SSIs participant, early 30s)

### Fast-track services for couples

Both male and female participants reported that health facilities in the district tend to prioritise antenatal care for women who bring their partners for ANC. Some men find this fast-tracking of services for couples appealing. Attending ANC was therefore perceived as a form of personal responsibility to facilitate care for their partners.

*It is well known that they [ ANC providers] always give priority to men. When you come with your husband, they always give you the privilege to be attended to first* (Female SSIs participant, late 20s)

While participants applauded the priority of services being accorded to couples, some however questioned its sustainability and fairness to women without partners.

*The next thing I was asking myself and my wife was what other feel about their marriages when they see us together at ANC? And if all the men decide to come, how will they treat the situation of fast-tracking services only for women who come with their partners? (*Male SSIs participant, early 30s)

## Discussion

Overall, our findings suggest that men in Bamenda ground their role to what is aligned with the cultural construction of masculinity and power while women align expectations to pragmatic support. Specifically, our analysis identified three categories of roles assumed by men during pregnancy in Bamenda: firstly breadwinning/financial provision for maternal nutrition and birth supplies was the most prominent role and echoed as the culturally sanctioned responsibility of men. Secondly, physical protection and psychosocial comfort was mentioned as the demonstration of shared partnership to protect women from the physical exhaustion of pregnancy and psychological distress. Thirdly, ‘sending’ women for ANC was mentioned as the extent to which men associate their involvement in pregnancy care because of the cultural delineation of roles that assign antenatal attendance to women and financial responsibility for ANC care to men. Beyond the ‘sender’ role, respondents advanced the following benefits for male ANC: direct access to expert MCH education, shared responsibility for pregnancy, paternal bonding, social protection and the appeal to the fast-track route for couple-focused ANC services.

Based on our study, the construction of male role and perceived involvement in pregnancy care in Bamenda, Cameroon is at the intersection of gender and socio-cultural norms on masculinity. This finding corroborates previous evidence across sub-Saharan Africa that reflect the dichotomy of ANC attendance as the preserve of women while financial provision for antenatal care is the responsibility of men [47–50]. A novel finding that emerged in our study was male perceptions that men participate indirectly in antenatal care through the finances they generate to send women for ANC and these finances in turn act as their representatives in antenatal clinics. This was mostly echoed by male participants who affirmed the need for male ANC attendance but distanced themselves from accompanying their partners on the basis of the need to engage in income-generating activities. The perspectives of our participants point not only to the primacy of male provision for the sake of maternal nutrition but also to the endorsement of the provider role as a means to fulfil the cultural identity of manhood. This falls in line with symbolic interactionist theory in which Blumer observed that individuals moderate their behaviours and aspire to identity standards that are in synch with prevailing social norms [32]. For expectant fathers in Bamenda, a critical discourse analysis of our data suggests that the most dominant identity men adhere to during pregnancy is that of provider and ‘sender’ for antenatal care.

Female respondents in our study argued for greater male involvement beyond financial roles, which is in contrast to a study in Ghana where female participants affirmed breadwinning roles as sufficient [51]. While limited male involvement in the Ghanian study was perceived as a form of emancipation and self-actualisation, we previously reported that some women disapprove of male involvement in order to extort finances from their husbands and also to avoid being targets for negative stereotyping [29]. However, in this present study, female participants desired shared responsibility for pregnancy care and sought to secure their reputational identities through their partner’s attendance of ANC.

Beyond breadwinning roles, participants acknowledged that pregnancy is physically tasking and often destabilises women psychologically. The role of physical protector through the assumption of duties like domestic chores and psychosocial support through affirmation and comfort, were often cited as part of a man’s role during pregnancy. While the perception that pregnancy is a shared responsibility was recurrent among female participants and backed by evidence as an important role that could prevent maternal complications [52, 53], few men affirmed this. In this study, we observed that how men described their supportive roles were not congruent with the experiences of women. This finding when paired with other evidence highlights the fact that although men recognise the physiological burden of pregnancy, this does not necessarily translate to the provision of physical and psychosocial support [48]. This could be as a result of the fact that roles that involve domestic and emotional support are not a culturally sanctioned or associated with the performance of the dominant form of masculinity in Bamenda Grassfield communities.

It has been reported elsewhere that men with limited awareness and knowledge on maternal and health child are less likely to be involved in pregnancy care [29, 54]. We found that the desire to acquire pregnancy-related knowledge directly from expert providers was the greatest motivation for male ANC attendance. Contrary to other studies [47, 55], we found little evidence on companionship or couple HIV testing as motivating factors for male ANC attendance. In the Cameroonian context, most male respondents perceived ANC attendance as a platform to reinforce their power through direct access to knowledge from medical experts. Companionship and bonding were mainly the concern of women who capitalised on this as a demonstration of their husband’s supportive act of care/love which reinforced their reputational status and social security.

By attending antenatal care, women gain access to maternal and child health education knowledge men are not privy to. While we did not explore how pregnant women engage their partners in post ANC discussions, our data highlights the tension that arise when women attempt to challenge male-dominated power structures by initiating post-antenatal care discussions with their partners. Due to entrenched gender norms, this was largely perceived by some men as an attempt to instruct them—a threat to their masculine identities and power. Female-initiated maternal and child health education was therefore downplayed by men and labelled as ‘strange’ or exaggerated. This tension in pregnancy-related communication has been reported in other studies where only 24% of men in Tanzania for example, were comfortable discussing prenatal issues with their partners [54]. These findings suggest that although ANC attendance is generally perceived as a woman’s business, women do not have the agency to fully implement health education without men sanctioning the sources of health information. Furthermore, while it is expected that a woman attends ANC as one being sent by a man, the prevailing power structure in Bamenda restricts her from discussing or educating her partner regarding his role in pregnancy care. Consequently, couple ANC attendance therefore appealed to some men on the basis of the opportunity it gave them to bypass women and reinforce their power through direct engagement with providers –termed experts. This was visible in the intended use of their medical knowledge—to hold women accountable or ensure compliance with medical recommendations.

The fast-track and prioritization of couple antenatal services was reported as one of the approaches that appeal to men and motivate them to attend antenatal care. This approach to incentivize male involvement in pregnancy care and it’s unintended consequence has been reported in other settings [55, 56]. While some men in Bamenda questioned the sustainability and fairness of such approaches, they however capitalised on the privilege and power this gives them to facilitate ANC access for their partners.

### Study limitation

This study had some limitations. Our participants were purposively selected. So, findings may not be generalisable beyond the study population. However the diversity of study participants suggest that our results reflect the larger community. Additionally, social desirability bias might have influenced participant responses as we noted that reports on the execution of some roles by men did not match the experiences of women. This was however minimised through our approach on methodological triangulation and thematic saturation.

## Conclusion

In this paper we discuss how masculine ideals in Bamenda are negotiated or leveraged upon to situate male involvement in the prenatal context. To address gaps in male involvement, we suggest that the health system and intervention designers capitalise on male interest in pregnancy education by: engaging men directly, deconstructing and expanding culturally sanctioned perceptions of masculinity to encourage broader involvement in non-materialistic forms of male involvement like ANC attendance, domestic and psychosocial support. This will necessitate the restructuring of service delivery to accommodate male attendance without further reinforcing the unequal power relations between pregnant women and their male partners.

## Data Availability

.The datasets generated and/or analysed during the current study are in Emglish and are not publicly available due to confidentiality of the participants but will be considered from the corresponding author on case-by-case basis
